# Screening of Microbial Volatile Organic Compounds for Detection of Disease in Cattle: Development of Lab-scale Method

**DOI:** 10.1038/s41598-019-47907-w

**Published:** 2019-08-20

**Authors:** Devin L. Maurer, Christine K. Ellis, Tyler C. Thacker, Somchai Rice, Jacek A. Koziel, Pauline Nol, Kurt C. VerCauteren

**Affiliations:** 1 Iowa State University, Dept. of Agricultural & Biosystems Engineering, Ames, IA 50011 USA; 2 USDA-APHIS-WS-National Wildlife Research Center, Fort Collins, CO 80521 USA; 3 USDA-ARS, National Animal Disease Center, Mycobacterial Diseases, Ames, IA 50010 USA; 4 USDA-APHIS-WS-Wildlife Livestock Disease Investigations Team, Fort Collins, CO 80521 USA

**Keywords:** Ecological epidemiology, Agroecology, Medical and clinical diagnostics, Infectious-disease diagnostics, Microbiology techniques

## Abstract

The primary hurdle for diagnosis of some diseases is the long incubation required to culture and confirm the presence of bacteria. The concept of using microbial VOCs as “signature markers” could provide a faster and noninvasive diagnosis. Finding biomarkers is challenging due to the specificity required in complex matrices. The objectives of this study were to (1) build/test a lab-scale platform for screening of microbial VOCs and (2) apply it to *Mycobacterium avium paratuberculosis*; the vaccine strain of *M*. *bovis* Bacillus Calmette-Guérin; and *M*. *kansasii* to demonstrate detection times greater those typically required for culture. SPME-GC-MS was used for sampling, sample preparation, and analyses. For objective (1), a testing platform was built for headspace sampling of bacterial cultures grown in standard culture flasks via a biosecure closed-loop circulating airflow system. For (2), results show that the suites of VOCs produced by *Mycobacteria* ssp. change over time and that individual strains produce different VOCs. The developed method was successful in discriminating between strains using a pooled multi-group analysis, and in timepoint-specific multi- and pair-wise comparisons. The developed testing platform can be useful for minimally invasive and biosecure collection of biomarkers associated with human, wildlife and livestock diseases for development of diagnostic point-of-care and field surveillance.

## Introduction

Bovine tuberculosis (bTB) is a zoonotic disease of international public health, trade, agricultural, and wildlife management significance^1,2^. The disease is caused by *Mycobacterium bovis*, a member of the *Mycobacterium tuberculosis* complex^3–5^. It is estimated that in 2014, 9.6 million new cases human tuberculosis (hTB) and 1.5 million associated deaths occurred worldwide^6^, with the majority caused by *M*. *tuberculosis*; however, zoonotic tuberculosis is often under-reported. Muller *et al*. 2013 reported that approximately 10–37% of hTB cases may be caused by *M*. *bovis* infection, especially in developing countries where the prevalence of livestock bTB may reach 10–14%^7–12^.

A primary hurdle for mycobacterial disease diagnosis is the long incubation time required to culture and confirm the presence of mycobacteria in biological samples. Culture may take approximately eight weeks before final results can be confirmed. Several *in vitro* blood based tests (i.e., interferon-ɣ release assay) have been developed to confirm that an individual has been exposed to mycobacteria. However, these tests suffer from cross-reactivity with closely related non-tuberculous mycobacteria resulting in false positive test results. Development of technologies to reduce the time between the start of culture, detecting growth, and positively identifying the mycobacterial agent will improve both the diagnosis and appropriate treatment of infected humans, and eradication of bTB from wildlife and livestock.

In recent years increased attention has been given to the concept of using microbial volatile organic compound (VOC) emissions as “signature markers” (a.k.a. biomarkers) for faster, more economical, and noninvasive disease diagnosis in humans and animals. These VOC emissions may be collected from breath, blood, skin, urine, feces and other bodily secretions. Studies have identified potential VOC biomarkers related to multiple diseases such as cholera, cancer, diabetes, uremia, schizophrenia, asthma, liver disease, chronic lung disease, Pseudomoniasis, tuberculosis, and others^13–16^. Several methods of collecting and analyzing VOCs for potential diagnosis of *M*. *tuberculosis* and *M*. *bovis* have been described. Closed loop stripping analysis (CLSA) - gas chromatography- mass spectrometry (GC-MS) was used to detect VOCs from multiple strains of *M*. *tuberculosis* from cultures^17^, and select ion flow tube (SIFT)-MS has been used to measure VOCs present in the headspace of *M*. *bovis* BCG cultures^18^ and to measure VOCs in the breath of children with cystic fibrosis^14^, NH_3_ levels in human breath for *Helicobacter pylori* screening, and detect acetonitrile levels in smokers’ breath^19^.

Electronic ‘e-nose’ technology has been used for detection of VOCs present in sputum samples collected from humans infected with *M*. *tuberculosis*^14^, and VOCs from the headspace of cultures of *M*. *bovis* BCG and *M*. *smegmatis*^18^. ‘E-nose’ has also been reported capable of differentiating between *M*. *tuberculosis*, three other bacteria, and a control^19^, monitoring smokers’ habits by measuring breath CO, detecting *H*. *pylori* presence in association with chronic gastritis, and detecting N_2_O produced by respiratory inflammation^16^. Thermal desorption (TD)-GC-MS has been used to determine VOCs present in the headspace of *M*. *bovis* BCG cultures^18^, and cattle breath^20^; and solid-phase microextration (SPME)-GC-MS was used to determine biomarkers *M*. *tuberculosis* and *M*. *bovis* in cultures and *M*. *tuberculosis* in human breath^21,22^. Other methods that have been used to collect VOCs associated with diseases that could be utilized in the future for TB detection include ion mobility spectrometry (IMS)^14,23^, proton transfer reaction (PTR)-MS^14–16^, and laser spectroscopy^24^. Select ion flow tube-MS, ‘e-noses’, IMS, PTR-MS have the advantage of being fast and potentially mobile. The downside of these methods includes decreased sensitivity, and the inability to chemically identify or profile VOCs. GC-MS-based methods may be slower, more expensive, and the instrumentation is not typically mobile; however, they have the advantage of being able to reproducibly identify and profile known and unknown microbial VOCs at low concentration ranges^14,16^.

Solid-phase microextration is an attractive technology for collecting microbial VOCs due to its simplicity, ease of use, and ability to sample and pre-concentrate a wide range of potential target compounds. Dynamic headspace extraction of VOCs can increase mass transfer to the SPME fiber compared to static headspace extraction, thus, reducing sampling times while improving mass loading of the fiber^25,26^. In contrast, both CLSA and TD are capable of pre-concentrating VOCs but require extra equipment and are more labor intensive than SPME. Syhre *et al*.^21^ collected VOCs from seven mycobacterial and 16 other respiratory pathogen cultures using three different SPME fiber types; 100 µm polydimethylsiloxane (PDMS), 2 cm 50/30 µm divinylbenzene (DVB)/Carboxen/PDMS, and 70 µm Carbowax/DVB. The SPME fiber coated with 2 cm 50/30 µm DVB/Carboxen/PDMS was found to recover higher concentrations of all target VOCs. Other studies have utilized the 50/30 µm DVB/Carboxen/PDMS-type and Carboxen/PDMS-type SPME fiber coatings for microbial VOC collection in human and cattle breath samples^22,27,28^, in bovine nasals excretions^29^, and in bovine fecal excretions from cattle vaccinated with *M*. *bovis* challenge^30^. SPME has been also used for the *in-vivo* and *in-vitro* collection of rumen gases^31,32^, VOCs emitted from wildlife marking fluids^33,34^, decaying carcasses^35^.

The objectives of this research were to (1) design, build, and test a lab-scale dynamic VOC sampling platform specifically capable of simultaneous biosecure SPME collection of headspace VOCs emitted from controlled bacterial cultures and a media control; (2) apply this screening method to *M*. *avium paratuberculosis* (MAP); the vaccine strain of *M*. *bovis* Bacillus Calmette-Guérin (BCG); and *M*. *kansasii* cultures to demonstrate a proof-of-concept detection method that is faster than standard culture methods.

Our first working hypothesis was that by sampling the recirculating culture headspace air, we would be able to detect trace-levels of microbial VOCs early in the incubation process with minimal background interference. Our second hypothesis was that SPME sampling of microbial VOCs followed by GC-MS analysis would be suitable to detect differences in microbial VOCs emitted by different cultured strains of mycobacteria.

If successful, this proof-of-concept identification of VOC biomarkers would allow differentiation between the microbial agents prior to the eight weeks often required for cultured mycobacterial strain identification. The knowledge gained from this work could be directly applied for diagnosis of hTB and bTB, for detection and identification of VOC biomarkers produced by culture of other pathogenic bacteria, and as a reference library for pathogen-produced VOCs present in other samples such as breath, feces, urine, blood, and other biofluids.

## Results and Discussion

### Reducing interfering background VOCs in lab-scale testing platform

**Figure 1:**
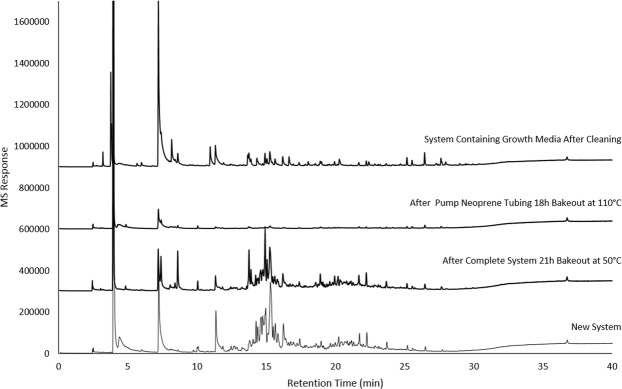
Minimization of interfering background VOCs from the lab-scale testing platform. Note: n = 3 for “after pump Neoprene tubing 18 h bake-out at 110 °C”, all others n = 4.

To minimize the VOC background and improve the likelihood of finding unique microbial VOCs for Objective 2 the prototype sampling platform was exposed to an initial 21 h bake-out at 50 °C, which only decreased the background slightly. After bake-out of the Neoprene tubing, system background emissions were reduced by 75% (Fig. 1). Background emissions present in the microbial growth media were also determined as reproducible and necessary background populated by numerous VOCs with relatively low peak areas.

### Multi-group analysis of single ion data

**Figure 2:**
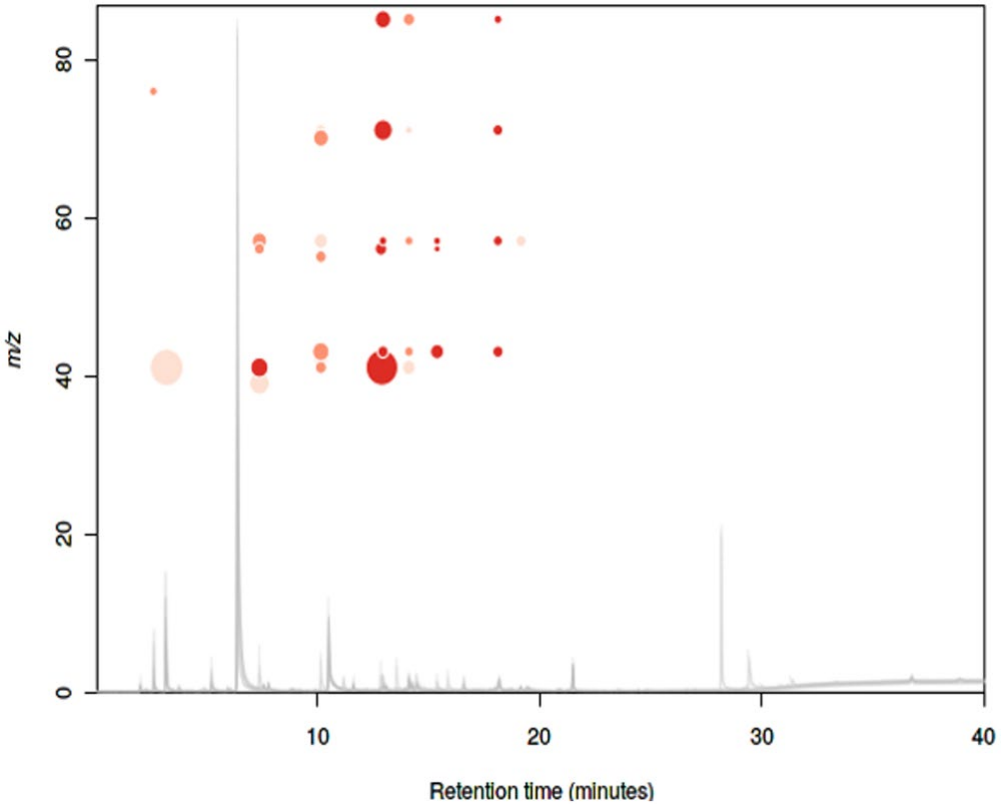
Cloud plot of 33 significantly different ions found in VOCs emitted from tested cultures. (Darker color = lower *p*-value; larger circle = greater fold change).

A total of 123 fragmentation ions were identified by XCMS Online, with 33 meeting the criteria for statistical significance (α = 0.05; fold change ≥1.5). Visualization of the statistically significant ion feature characteristics are depicted by XCMS Online as a cloud plot (Fig. 2)^36^. Briefly, all sample chromatograms were aligned and overlaid onto the x-axis. Significant ion features are identified as circles, with ions with greatest m/z ratios located furthest from the x-axis. Circle size is proportional to the degree of fold change (larger circle = greater fold change), while color intensity corresponds to the statistical significance (*p*-value) of the fold change as calculated by a Welch t-test with unequal variances (darker color = lower *p*-value).

### Identification of culture specific peak area data

**Figure 3:**
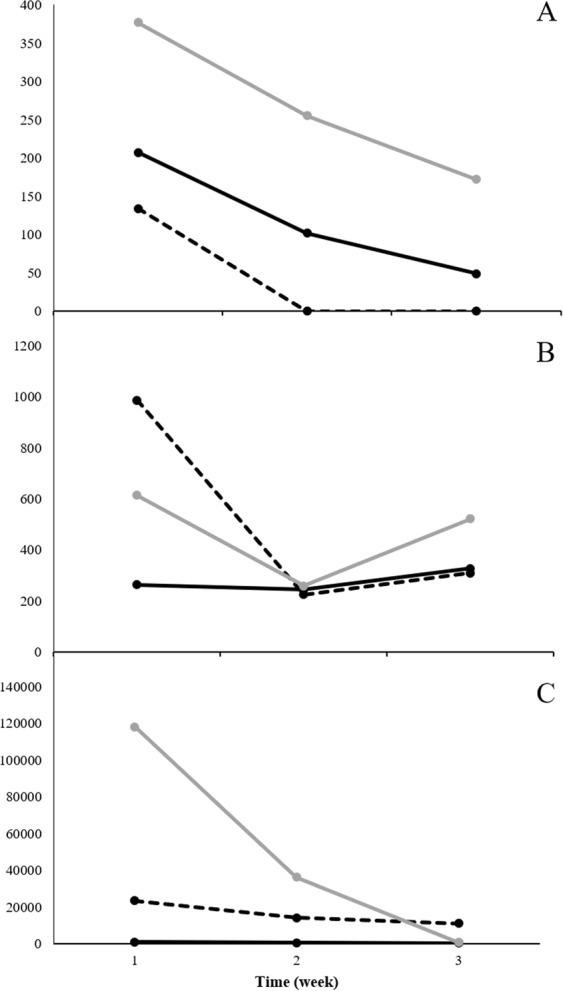
Weekly trends of three compounds found in *M*. *bovis* Bacillus Calmette-Guérin (BCG) cultures. Note: (**A**): Compound 1 identified at retention time 4.250 min, (**B**): Compound 2 identified at retention time 9.286 min, (**C**): Compound 3 identified at retention time 17.080 min. Black solid line: Replicate 1, Dotted black line: Replicate 2, Gray solid line: Replicate 3.

**Figure 4:**
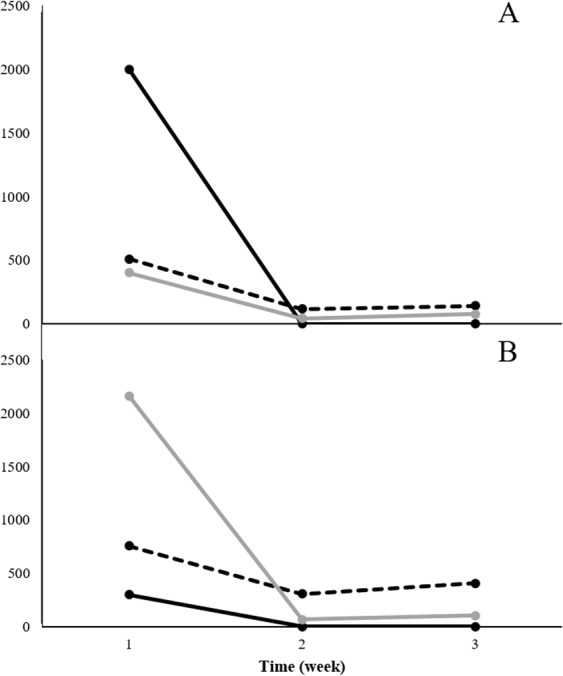
Weekly trends of two compounds identified in *Mycobacterium avium paratuberculosis* cultures. Note: (**A**): Compound 1 identified at retention time 5.309 min, (**B**): Compound 2 identified at retention time 9.286 min. Solid black line: Replicate 1, Dotted black line: Replicate 2, Solid gray line: Replicate 3.

**Figure 5:**
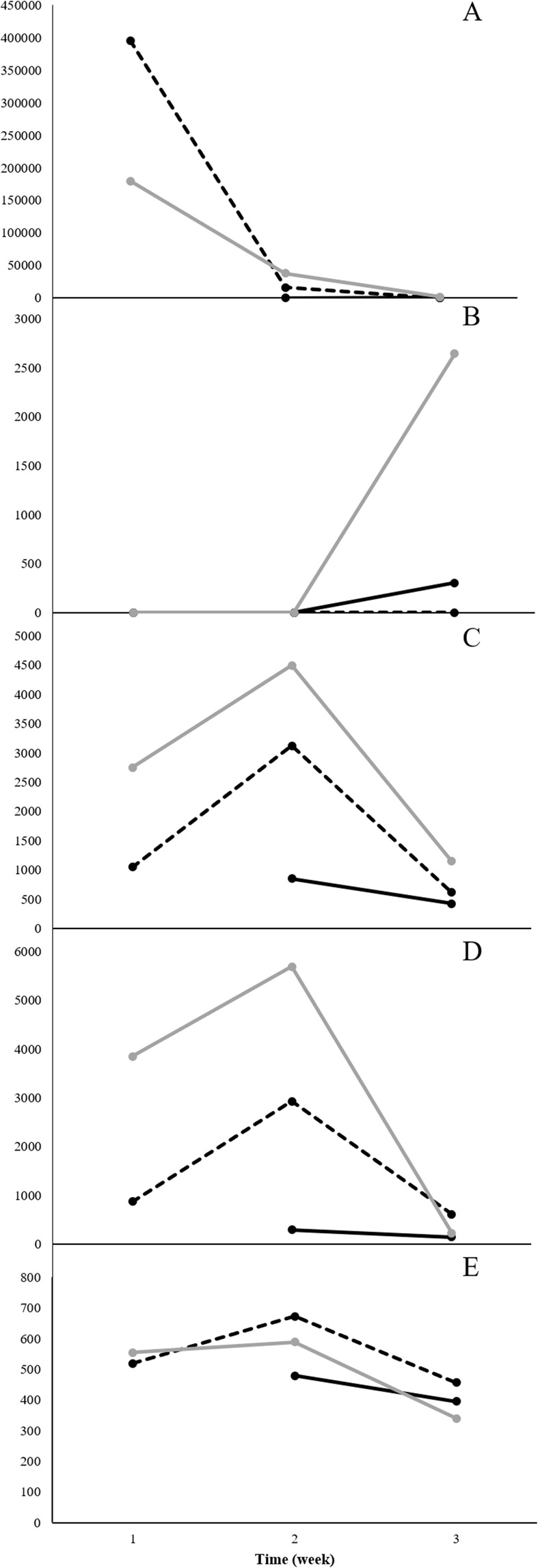
Weekly trends of five compounds identified in *M*. *kansasii* cultures. Note: (**A**): Compound 1 identified at retention time 5.309 min, (**B**): Compound 2 identified at retention time 11.344 min, (**C**): Compound 3 identified at retention time 18.769 min, (**D**): Compound 4 identified at retention time 19.048 min, (**E**): Compound 5 identified at retention time 20.114 min. Black solid line: Replicate 1, Dotted black line: Replicate 2, Solid gray line: Replicate 3.

Statistically significant ion intensities were matched by retention time to 77 total ion chromatographic (TIC) peaks. For each culture, trends in the changes of potential VOCs TIC intensities across the three time-points in each of the replicates were graphed. This allowed identification of ten VOC compounds with consistent, repeatable changes across time in each of the three replicates (3 studies) (Figs 3, 4 and 5).

### Tentative identification of biomarkers

Tentative identifications of peaks and associated information are summarized in Table 1. Tentatively identified compounds include two aldehydes (octanal, decanal); two alkanes (3,3-dimethyl hexane, tetradecane); three benzenes (benzene, ethylbenzene, styrene); one dicarboxylic acid (pentanedioic acid, 2,4-dimethyl dimethyl ester); one isothiocyanate (cyclohexane, isothiocyanate); two ketones (2-pentanone, acetophenone); and one oxotane (lilac aldehyde B). Two unique (benzene, acetophenone) and one shared (ethylbenzene; with MAP) are identified in BCG cultures. Two shared compounds (2-pentanone, *M*. *kansasii*; ethylbenzene, BCG) are associated with MAP cultures, while four unique (styrene; pentanedioic acid, 2, 4-dimethyl, dimethyl ester; 3,3-dimethyl hexane; cyclohexane, isothiocyanate) and one shared compound (2-pentanone, MAP) are associated with *M*. *kansasii* cultures. Four unique compounds (octanal, decanal, tetradecane, lilac aldehyde D) appear in the control media.

**Table 1:** Compounds allowing discrimination among three mycobacterial cultures and control media at three weekly time-points.

Mean Retention Time (min)	BCG	MAP	Control	*M*. *kansasii*	Compound	Five Most Abundant Ions/Relative Abundance
4.250	X				Benzene	78/999, 77/283, 51/221, 50/208, 52/118
5.309		X		X	2-Pentanone	43/999, 89/197, 41/138, 58/98, 71/91
9.286	X	X			Ethylbenzene	91/999, 106/282, 51/114, 65/113, 77/99
11.344				X	Styrene	104/999, 103/485, 78/464, 51/267, 77/219
13.134			X		Octanal	48/999, 44/808, 41/670, 56/657, 84/550
17.788	X				Acetophenone	105/999, 77/868, 51/378, 120/204, 43/179
18.333			X		Decanal	43/999, 41/807, 57/621, 55/618, 44/539
18.769				X	Pentanedioc acid, 2, 4-dimethyl, dimethyl ester	69/999, 59/878, 128/814, 41/541, 73/480
19.048				X	3,3-dimethyl hexane	43/999, 57/636, 71/613, 85/501, 41/443
20.114				X	Cyclohexane, isothiocyanate	55/999, 83/609, 41/531, 141/528, 82/284
21.495			X		Tetradecane	57/999, 43/740, 71/643, 85/423, 41/261
21.743			X		Lilac aldehyde D	55/999, 43/724, 41/532, 71/330, 93/317

*Mycobacterium bovis* Bacillus Calmett- Guérin (BCG) cultures contain two unique and one shared VOCs that allow good discrimination from the other cultures and control media. *M*. *avium paratuberculosis* (MAP) cultures are identified using two shared VOCs. *M*. *kansasii* is identifiable using four unique and one shared VOC. Culture media is discriminated from the cultures using four VOCs.

The four compounds unique to the control media were removed from further evaluation, leaving ten mycobacterial-associated compounds to evaluate for potential bacterial metabolic or physiologic associations (Table 2).

**Table 2:** Potential cellular and metabolic sources of VOCs in three mycobacterial cultures.

Mean Retention Time (min)	Mycobacterial culture	Compound	Potential Cellular and Metabolic Associations
3.681	*M*. *kansasii*	Ethanol	Metabolized into acetyl CoA, used for energy in the citric acid cycle. Can be converted to acetaldehyde and then into acetic acid. Small amounts are endogenously produced via anaerobic fermentation^43^. Identified previously in MAP cultures^48^.
4.250	BCG	Benzene	Identified previously in *M*. *tuberculosis* cultures^17^, and MAP^48,49^.
5.309	*M*. *kansasii* MAP	2-pentanone	Identified in breath and feces of MAP infected goats^50^. 3-pentanone and methyl isopropyl ketone are isomer^43^.
9.286	BCG MAP	Ethylbenzene	Metabolite formed during degradation of styrene^40^. Some mycobacterial strains have been demonstrated capable of ethylbenzene degradation^51^^,^^52^. Identified previously in breath samples of *M*. *tuberculosis* infected humans^53^.
11.344	*M*. *kansasii*	Styrene	*M*. *tuberculosis* is capable of degrading styrene under hypoxic conditions which may play a role in intracellular survival^54^. Identified previously in breath samples of *M*. *tuberculosis* infected humans^53^.
13.354	*M*. *kansasii*	Octanal	A substrate for fatty aldehyde dehydrogenase and alcohol dehydrogenase^43^. Mycobacteria have been shown to utilize alcohol dehydrogenases in the biosynthesis of cell envelope lipids^55,56^.
17.788	BCG	Acetophenone	By-product of ethylbenzene metabolism^43^ Has been identified different concentrations in feces of white-tailed deer (*Odocoileus virginianus*) vaccinated with BCG or infected with *M*. *bovis*^57^.
18.769	*M*. *kansasii*	Pentanedioic acid, 2,4-dimethyl, dimethyl ester; (dimethyl glutarate)	
19.048	*M*. *kansasii*	3,3-dimethyl hexane	
20.114	*M*. *kansasii*	Cyclohexane, isothiocyanate	Isothyocyanates are formed by enzymatic conversion of glucosinolates which are synthesized from amino acids^58^

Potential cellular and metabolic sources of tentatively identified VOC compounds were identified for *Mycobacterium bovis* Bacillus Calmett- Guérin (BCG), *M*. *avium paratuberculosis* (MAP) and *M*. *kansasii* cultures.

There has been little research exploring the metabolic pathways of *M*. *bovis* BCG, MAP, and *M*. *kansasii*, therefore, published literature exploring the metabolome of *M*. *tuberculosis* complex and environmental strains of mycobacteria were considered sources for comparison (Table 3).

**Table 3:**
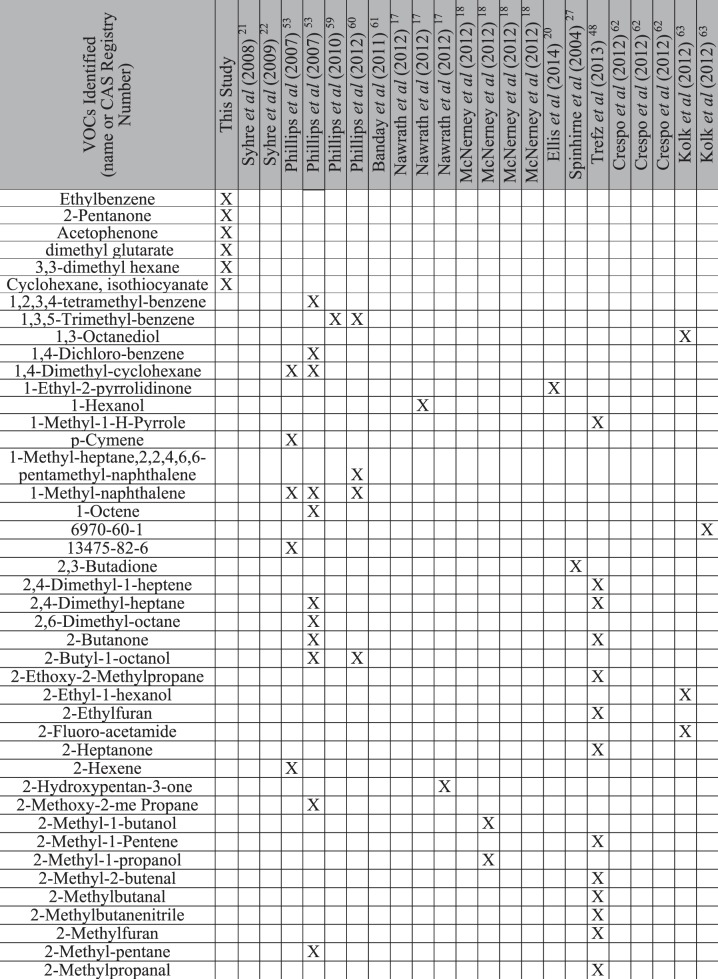

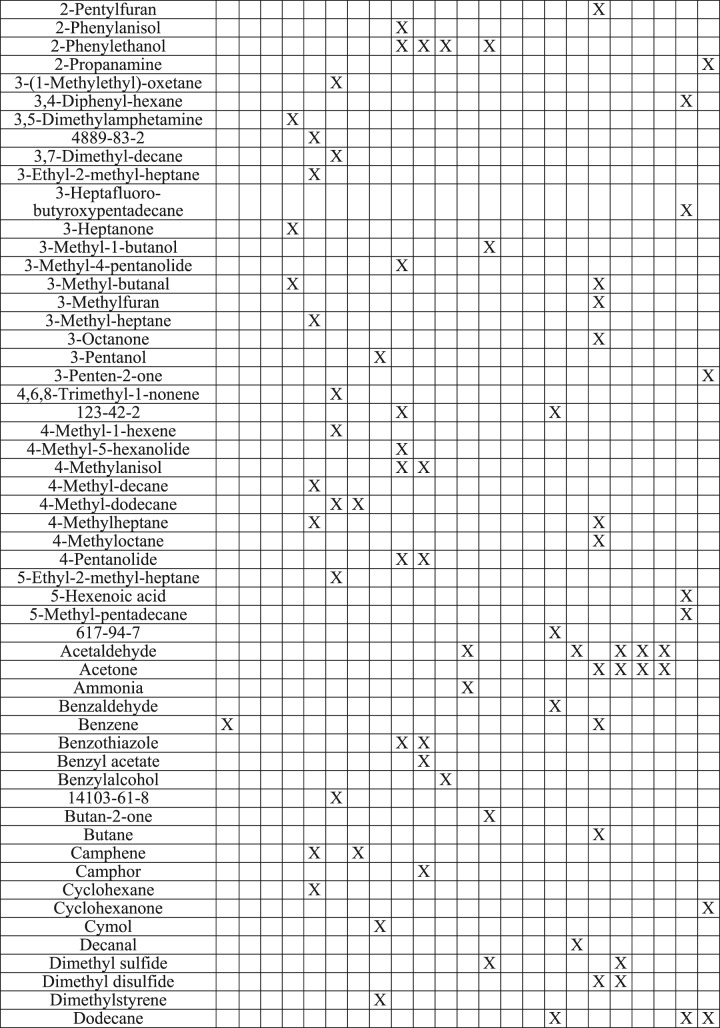

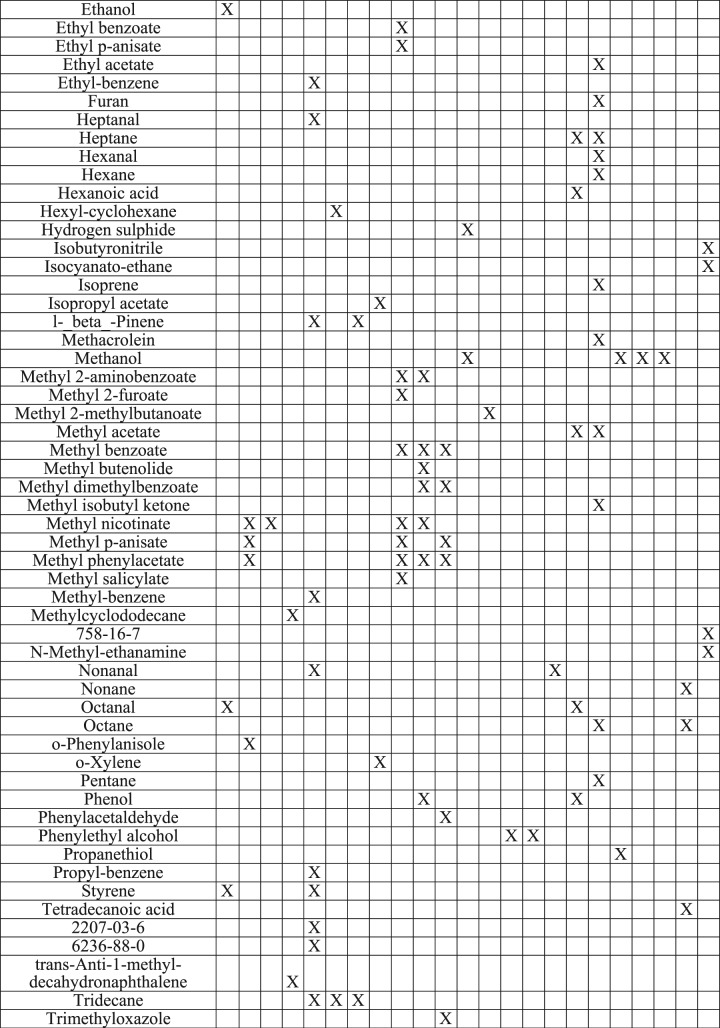

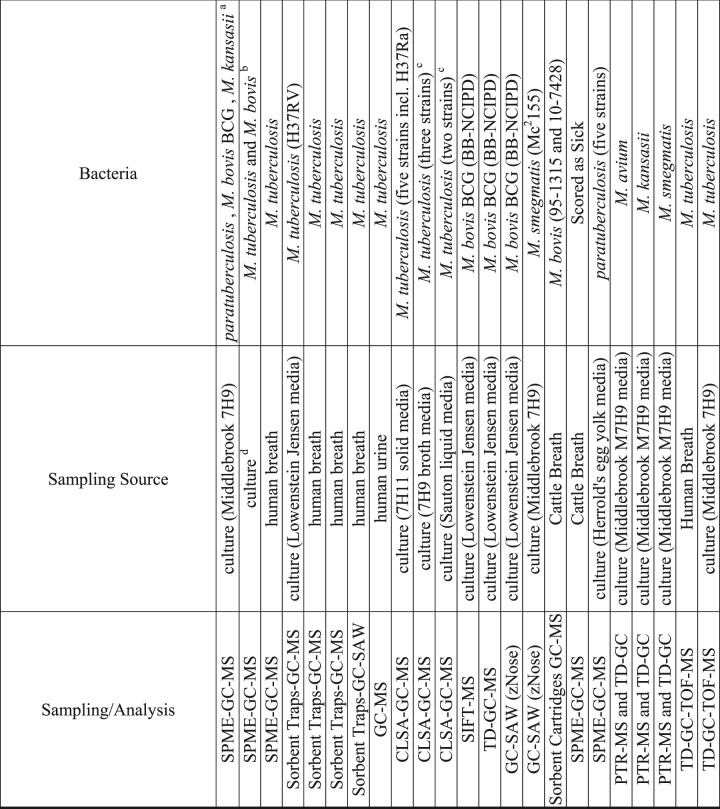
Comparison of VOCs identified in this study with those reported in the literature as associated with various bacteria in cultures, human and cattle breath^17,18,20–22,27,48,53,59–63^.

Note: ^a^*Paratuberculosis* (1331), *M*. *bovis* BCG (K10), *M*. *kansasii* (03-6931).

^b^*M*. *tuberculosis* (H37Ra) and *M*. *bovis* (NCTC 10772); Also reported on BCG (Danish strain 1331), *M*. *fortuitum* (NCTC 10394), *M*. *chelonae* (NCTC 946), *M*. *abcsessus* (TMC 1542), and at least five strains each of *A*. *fumigatus*, *A*. *flavus*, *A*. *niger*, *A*. *terreus*, *Fusarium spp*., *P*. *Aeruginosa*, *Rhizopusarrhizus*, *S*. *apiospermum*, *C*. *albicans*, *Burkholderiacepacia*, *P*. *Fluorescens*, *Staphylococcusaureus*, *E*. *coli*, *S*. *pneumoniae*, *Moraxellacatarrhalis*, and *H*. *influenza*. Only reported VOCs that were distinctive to *M*. *tuberculosis* &*M*. *bovis*.

^c^Also reported on non-TB mycobacteria: *Nocardia spp*, *N*. *africana*, *M*. *smegmatis*, *M*. *aurum*, *M*. *neoaurum*, *M*. *aichiense*, *M*. *scrofulaceum*, *M*. *avium ssp*. *avium*, *M*. *vaccae* not reported here.

^d^Culture (Lowenstein Jensen/glycerol, sheep blood agar and BacT/Alert MP media).

## Conclusions

This study demonstrates a proof-of-concept for the detection and use of microbial VOCs as a means to discriminate between mycobacterial cultures associated with one to three week post-culture inoculation, a time span preceding the time required to currently identify pathogenic *M*. *tuberculosis* and *M*. *bovis* in diagnostic cultures. To accomplish this task (Objective 1), a sampling system was designed, built, and tested for controlled collection of microbial volatiles using closed-loop headspace airflow over microbial cultures, sampling and sample preparation with SPME, analyte separation and identification via GC-MS, and tentative compound identification using novel metabolomics databases and the NIST W8N08 library. The capability of this system to produce results more efficiently than some currently utilized diagnostic modalities such as culture exemplifies its potential use as a diagnostic tool. The lab-scale testing platform concept can be useful for minimally invasive and biosecure collection of marker volatiles associated with human, wildlife and production animal diseases for development of diagnostic non-invasive point-of-care tools, field surveillance technologies and strategies.

Objective 2 provided a comprehensive assessment of VOCs collected from the headspace of three different mycobacterial cultures and one control media sample. Discrimination between mycobacterial cultures was successful one, two, and three weeks post-culture inoculation, a time span preceding the time required to currently identify pathogenic *M*. *tuberculosis* and *M*. *bovis* in diagnostic cultures. Unique VOCs representing potential biomarkers were identified in two mycobacterial cultures (e.g., BCG, *M*. *kansasii*). No unique plausible biomarkers were identified for the MAP cultures; however, discrimination from the two other mycobacterial cultures was possible when the VOC profiles of all the cultures were examined in context.

From a diagnostic perspective, detection of VOCs produced by pathogenic mycobacteria at early states of culture growth could improve disease diagnosis and treatment, especially in developing countries where access to sophisticated laboratory diagnostics is limited. The capability to differentiate between human and zoonotic mycobacteria under such circumstances could improve the capability of physicians to more accurately diagnose tuberculosis patients, to differentiate between hTB and zoonotic bTB, and to appropriately dispense medication targeted toward the etiological disease agent.

## Materials and Methods

### Experimental design

The experimental part of this study was carried out at the USDA-ARS National Animal Disease Center (NADC) and the Atmospheric Air Quality Laboratory of Iowa State University (ISU) in accordance with the Guide for the Institutional Animal Care and Use Committee. The protocol was approved by Iowa State University’s Institutional Animal Care and Use Committee (IACUC Log # 4-14-7787-B) and Institutional Biosafety Committee (ID: 14-I-015-A/H).

The proof-of-concept study was designed to determine variations and identify specific VOCs produced by growing mycobacterial cultures. Mycobacterial strains included *M*. *avium paratuberculosis (MAP;* Strain K10); *M*. *bovis* Bacillus Calmette-Guérin (BCG) (Danish 1331); and *M*. *kansasii* (Strain 03-6931). Approximately one optical density of each mycobacterial strain was added to respective 225 mL (culture surface area, 75 cm^2^) culture bottles (430725, Corning®, Corning, New York, USA) containing 30 mL of Middlebrook 7H9 media enriched with 10% Middlebrook OADC, 0.05% Tween-80 and 2 mg mL^−1^ of Mycobactin J. Cultures were incubated at 37 °C on the testing platform in an incubator for three weeks. Dynamic headspace samples of each culture bottle were collected weekly with SPME. A control consisting of only growth media was used to determine “background” VOCs.

### Lab-scale testing platform

**Figure 6:**
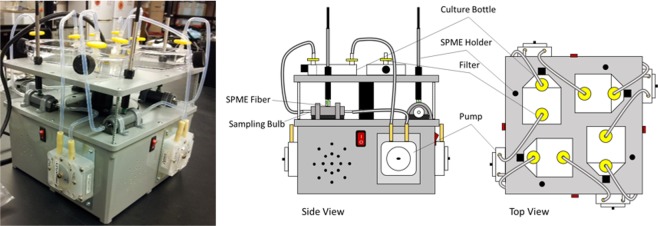
Lab-scale testing platform for biosecure collection of microbial VOCs with SPME.

The lab-scale testing platform was designed, built and tested using several general lab use components. The reusable testing platform consisted of four Omegaflex FPU100 peristaltic pumps (Omega Engineering Inc., Stamford, CT, USA) to circulate air (average flow rate of 162 mL min^−1^) through each modified culture bottle in closed loops with each separate loop containing an inline ~13 mL, glass sampling bulb (28526-U, Supelco, Bellefonte, PA, USA) for SPME extraction (Fig. 6). Each closed loop was constructed from PTFE Teflon tubing from the pumps to the culture bottles, to the sampling bulbs, back to the pumps; and Neoprene tubing sections in the peristaltic pumps. The inlet and outlet fittings on each culture bottle included 0.22 micron filters to prevent introduction of mycobacteria into the closed loop system and rendering the system to be self-contained and biosecure. The platform’s compact size enabled the entire system to be placed in an incubator for optimal temperature for growing mycobacteria. The materials used to constuct the platform allowed relatively easy decontamination after and between-the-trials use.

### Microbial volatile organic compounds

A 2 cm 50/30 µm DVB/Carboxen/PDMS fiber (57348-U, Supelco, Bellefonte, PA, USA) SPME fiber was used to extract and pre-concentrate VOCs from circulating gas. Samples were collected by dynamic headspace extraction with SPME for 1 h at 37 °C using the lab-scale testing platform. After the sampling period, the SPME fiber with extracted VOCs was inserted into the 250 °C GC injector for 2 min for thermal desorption, sample introduction, analyte separation and analysis.

The multidimensional GC–MS-Olfactometry (MDGC-MS-O) system (Microanalytics, Volatile Analysis Corporation, Round Rock, TX, USA) used for analysis was equipped with two columns connected in series. The non-polar pre-column was 30 m × 0.53 mm i.d.; film thickness, 0.50 µm with 5% phenyl polysilphenylene siloxane stationary phase (SGE BPX-5) and operated with constant pressure mode at 13.5 psi (0.92 atm). The polar analytical column was 30 m × 0.53 mm bonded polyethylene glycol (PEG) embedded in a synthetic glass (SGE SolGel-Wax) at a film thickness of 0.50 µm. System automation and data acquisition software were MultiTraxTM V. 10.1 (Microanalytics, Volatile Analysis Corporation, Round Rock, TX, USA) and ChemStation™ (Agilent Technologies, Santa Clara, CA, USA). The GC run parameters were as follows: injector, 250 °C; column, 40 °C initial, 3 min hold, 7 °C min^−1^ ramp to 240 °C final, 8.43 min hold; carrier gas, UHP-grade helium (99.999%). The GC was operated in a constant pressure mode where the mid-point pressure, i.e., pressure between pre-column and analytical column, was always at 5.7 psi (0.39 atm) and the heart-cut sweep pressure was 5.0 psi. This type of columns configuration (in series with different polarity) does not lend itself to a classic retention index (RI) approach for tentative compound identification with n-alkanes. The MS full scan range was 34 to 150 m z^−1^. Spectra were collected at 2 scans s^−1^ using full scan. The quadrupole MS was set to electron ionization (EI) mode with ionization energy of 70 eV. MS tuning was performed using the default autotune setting using perfluorotributylamine (PFTBA) daily.

### Reducing interfering background VOCs in lab-scale testing platform

After construction, the platform’s background VOCs were initially baked-out for 21 h at 50 °C (the maximum temperature that some plastic components in the platform could withstand) The Neoprene tubing in the peristaltic pumps was removed from the platform and baked-out separately at 110 °C for 18 h to remove any VOCs the might be released into the closed platform loop.

### Data analysis

Cultures were identified in raw data as culture 1–4 and sample collection time-point (Week 1, Week 2, Week 3) until the data analysis was complete to prevent bias. Total ion chromatograms (TICs) from all cultures at all time-points were analyzed using the multi-group comparison feature in XCMS Online to identify peak ion abundances that differed between the cultures at each time-point^37^. Statistically significant ion intensities were matched to GC column retention time chromatographic peaks using Agilent Mass Hunter software (Agilent Technologies, Santa Clara, CA, USA). Peak areas were determined using the TICs. Peak area data for each culture at each replicate and time-point were evaluated to determine the suite of VOCs best suited to provide optimal discrimination in multi-group and pair-wise comparisons. Peaks were tentatively identified using AMDIS deconvolution software^38^, the National Institute of Standards and Technology (NIST) W8N08 database^39^, the Kyoto Encyclopedia of Genes and Genomes (KEGG) database^40–42^, and the Human Metabolome Database (HMDB)^43–46^. Tentative compound identifications were made based on ≥65% match with compounds present in these libraries. Tentative metabolic sources for each compound were explored using KEGG, HMDB, and review of peer-reviewed literature^47^.
